# Demonstrative Midwifery

**Published:** 1850-11

**Authors:** 


*Demonstrative Midwifery.*—The October number of the American Journal of Medical Sciences contains an article over the signature of C. M., purporting to be a review of the Report of the trial of the People vs. Loomis, in which the writer animadverts on the case of Demonstrative Midwifery at the Buffalo Medical College during the session of 1849–50. As a solitary instance in which that subject has been discussed, in a medical Journal, by one totally opposed to the plan proposed and exemplified by the Professor of Obstetrics, and Faculty of the Buffalo College, we have every disposition to transfer the article to the Eclectic Department of our Journal. We have not done so solely because the subject has already engrossed a considerable share of our columns of late, but upon receiving an intimation that it will be acceptable to a fair proportion of our readers, or that it is a matter of justice to the opponents of demonstrative midwifery, we will give the article an insertion in our next number. While the opinions of any who may choose to take exceptions to an expression of our views on this, or any other topic, with as much freedom and frequency as seems to us proper, will not give us much concern, provided we are unconscious of indulging in personalities or any other breach of decorum, we desire to consult the interests and tastes of our readers so far as to avoid indulging a tedious partiality for subjects which may chance to engage our attention.

The article referred to, we understand, in this region is currently reported to be from the pen of Prof. C. D. Meigs. This, however, is assuredly an error, which it is but due to Professor M. should be corrected. The author is presumed to be Dr. Caspar Morris, of Philadelphia.

